# Proximity to Traffic, Inflammation, and Immune Function among Women in the Seattle, Washington, Area

**DOI:** 10.1289/ehp.11580

**Published:** 2008-10-16

**Authors:** Lori A. Williams, Cornelia M. Ulrich, Timothy Larson, Mark H. Wener, Brent Wood, Peter T. Campbell, John D. Potter, Anne McTiernan, Anneclaire J. De Roos

**Affiliations:** 1 Department of Epidemiology, School of Public Health and Community Medicine, University of Washington, Seattle, Washington, USA; 2 Cancer Prevention Program, Division of Public Health Sciences, Fred Hutchinson Cancer Research Center, Seattle, Washington, USA; 3 Department of Civil and Environmental Engineering, College of Engineering, University of Washington, Seattle, Washington, USA; 4 Department of Laboratory Medicine; 5 Department of Medicine, School of Medicine, University of Washington, Seattle, Washington, USA; 6 Program in Epidemiology, Division of Public Health Sciences, Fred Hutchinson Cancer Research Center, Seattle, Washington, USA

**Keywords:** air pollution, C-reactive protein, cytotoxicity, immune function, inflammation, lymphocyte proliferation, natural killer cell, traffic

## Abstract

**Background:**

Traffic-related air pollution has been associated with adverse health outcomes, and the immune system may be a biologic mediator of health effects.

**Objectives:**

We analyzed associations between living near major roads and immune status as measured by five immune assays. We hypothesized that living near a freeway, arterial, or truck route would be associated with increased inflammation and decreased immune function.

**Methods:**

We used a geographic information system (GIS) to determine residential proximity to major roads among 115 postmenopausal, overweight women in the greater Seattle, Washington (USA), area whose immunity was assessed at the baseline visit of an exercise intervention trial. We evaluated three inflammatory markers (C-reactive protein, serum amyloid A, and interleukin-6) and two functional assays of cellular immunity [natural killer (NK) cell cytotoxicity and T-lymphocyte proliferation].

**Results:**

Women living within 150 m of arterial roads had 21% lower NK cytotoxicity compared with women who lived farther from an arterial [mean cytotoxicity, 19.5%; 95% confidence interval (CI), 15.6–23.5%; vs. mean cytotoxicity, 24.8%; 95% CI, 22.0–27.5%], after adjustment for both individual-level and census tract–level demographic characteristics. This association was limited to women who reported exercising near traffic. Fewer women lived near freeways and truck routes. Markers of inflammation and lymphocyte proliferation did not consistently differ according to proximity to major roads.

**Conclusions:**

If the observed association between residential proximity to traffic and decreased NK cytotoxicity is confirmed in other populations, our results may have implications for local land use policy.

Previous studies of traffic-related pollution have observed associations between motor vehicle pollution and an array of adverse health outcomes, including respiratory and cardiovascular diseases (White et al. 2005). The immune system is a hypothesized biologic mediator of such health effects (Brook et al. 2004; Devalia et al. 1997). The strongest evidence of an association between air pollution and immune status in adults comes from studies of serum levels of C-reactive protein (CRP)—an acute-phase reactant and well-established marker of inflammation—in which indicators of increased air pollution were associated with higher CRP levels (Delfino et al. 2008; Diez Roux et al. 2006; Dubowsky et al. 2006; Peters et al. 2001; Pope et al. 2004; Riediker et al. 2004; Ruckerl et al. 2006; Seaton et al. 1999; Yue et al. 2007; Zeka et al. 2006). The hypothesis that air pollution may cause immune-suppressive effects is supported by animal studies demonstrating increased susceptibility to infection with diesel exhaust exposure [U.S. Environmental Protection Agency (EPA) 2002] and epidemiologic studies showing increases in hospital admissions for respiratory infections with increases in nitrogen dioxide among the general population (Fusco et al. 2001; Lin et al. 2005). None of the previous studies of air pollution and immune function in adults has directly evaluated exposure to traffic.

We studied the association between traffic-related pollution and biomarkers of systemic inflammation and cellular immunity in the Puget Sound region of Washington State. Because traffic-related pollutants, such as nitrogen dioxide and ultrafine particles, are high near major roadways and then decay exponentially over a short distance (Lebret et al. 2000; Roorda-Knape et al. 1999; Zhu et al. 2002), we assessed exposure according to residential proximity to major roads. Our study population consisted of overweight, postmenopausal women, a group that may be particularly vulnerable to air pollution–related health effects, based on results of previous studies showing the strongest air pollution associations with inflammatory markers among obese persons (Dubowsky et al. 2006; Zeka et al. 2006).

We investigated three markers of systemic inflammation—CRP, serum amyloid A (SAA), and interleukin-6 (IL-6)—and two measures of cellular immunity, natural killer (NK) cell cytotoxicity and T-lymphocyte proliferation. CRP is a recognized predictor of cardiovascular disease, and SAA and IL-6 may also predict inflammation-related diseases (Ershler 1993; Johnson et al. 2004; Kritchevsky et al. 2005; Yeh and Willerson 2003). The NK cytotoxicity assay measures the ability of NK cells to kill cancerous target cells (Albers et al. 2005; Vedhara et al. 1999). Low levels of NK cytotoxicity are believed to reflect a defect in the natural immune response and may predict risk of future adverse health events, including infection and cancer (Imai et al. 2000; Levy et al. 1991; Mizutani et al. 1996; Ogata et al. 2001). Higher levels of *in vitro* lymphocyte proliferation are believed to reflect a more effective immune response (Albers et al. 2005; Imai et al. 2000; Levy et al. 1991; Ogata et al. 2001; Vedhara et al. 1999).

## Materials and Methods

### Study design

We conducted a cross-sectional analysis of the associations between traffic-related pollution and a set of five immune assays using data from the baseline visit of an intervention trial of exercise conducted at the Fred Hutchinson Cancer Research Center (FHCRC) (McTiernan et al. 1999). In our study procedures, we complied with all applicable U.S. requirements (including the FHCRC and University of Washington institutional review boards), and all women gave written informed consent before participation in the study.

### Study population

Women were recruited from the greater Seattle area from 1998 to 2000 to participate in the Physical Activity for Total Health study, a 12-month randomized controlled intervention trial comparing the effects of a moderate-intensity exercise intervention versus a stretching control program on endogenous sex hormones in postmenopausal women (McTiernan et al. 1999). Subjects in a substudy of immune function (*n* = 115) were women who also met criteria for measurement of immunologic outcomes. Women were 50–75 years of age, were nonsmokers, consumed fewer than two alcohol drinks per day, were sedentary, were overweight or obese [body mass index (BMI) ≥ 25.0; or between 24.0 and 24.9 with percentage body fat > 33%], were postmenopausal and not taking hormone replacement therapy in the preceding 6 months, and had no history of invasive cancer, diabetes, cardiovascular disease, or asthma; additional eligibility criteria have been published previously (McTiernan et al. 1999). Women in the substudy of immune function were eligible if they had no current serious allergies, were not regular (two or more times/week) users of aspirin or other nonsteroidal anti-inflammatory medications, and were not using corticosteroids or other medications known to affect immune function (Shade et al. 2004).

### Questionnaires and interviews

Information on demographics (age, education, income, employment status, marital status, and race/ethnicity), smoking history, and exercise was collected via a self-administered questionnaire. Body height and weight were measured during a clinical exam using a standard protocol. Use of multivitamins was determined from an in-person interview with each subject where supplement labels were photocopied and data were abstracted.

### Immune measures

All women came to the University of Washington Department of Laboratory Medicine for blood draws. Twelve-hour fasting blood samples were taken between 0730 and 0830 hours following strict blood-draw criteria described previously (Boynton et al. 2007). Serum and plasma were processed within 1 hr of collection and stored at –70°C. All immune assays were conducted at the University of Washington Clinical Immunology Laboratory in the Department of Laboratory Medicine.

We measured serum CRP and SAA by latex-enhanced nephelometry using high- sensitivity assays on the Behring Nephelometer II analyzer (Dade-Behring Diagnostics, Deerfield, IL) with lower detection limits of 0.2 mg/L for CRP and 0.7 mg/L for SAA. The interassay (between-batch) coefficients of variation (CVs) were 5–9% for CRP and 4–8% for SAA.

For serum IL-6, we performed solid-phase sandwich enzyme-linked immunosorbent assays with the Biosource Human IL-6 Immunoassay kit (Biosource, Camarillo, CA). The interassay CVs were < 10% for concentrations > 35 pg/mL, and analytical sensitivity was 8 pg/mL.

Our method for measuring NK cytotoxicity using a flow-cytometric assay has been described previously (Shade et al. 2004). NK cells were isolated from peripheral blood mononuclear cells (PBMCs) obtained from 14 mL heparin-anticoagulated blood by Ficoll-Hypaque separation. We washed and diluted cells to a mononuclear cell concentration of 7.7 × 10^6^ cell/mL.

We prepared K562 target cells as follows: We washed cells in the log phase of growth twice and incubated them with label 3,3′-dioctadecyloxa carbocyanine perchlorate (DiO; Live/Dead cytotoxicity kit no. L7010; Molecular Probes, Eugene, OR). We incubated, washed, and resuspended the cells to a concentration of 1 × 10^6^ cells/mL and then filtered them through a 35-μm strainer.

We serially diluted the culture-suspended NK cells to four effector-to-target cell (E:T) ratios of 50:1, 25:1, 12.5:1, and 6.25:1 and then pelleted and incubated the cells. We added propidium iodide to a final concentration of 0.03 mg/mL and transferred the cells to a polypropylene tube for flow cytometric analysis to identify dead cells. We used the percentage of dead target cells among a total DiO-identified target cells as the measure of NK cytotoxicity. We performed each assay in duplicate and with appropriate controls. Within-run CVs ranged from 5.9% to 8.9%.

We assessed T-lymphocyte proliferation using cryopreserved PBMCs with two methods: ^3^H-thymidine incorporation in response to the mitogen phytohemagglutinin (PHA), and the cell-division tracking method in response to anti-CD3 antibody. We have described these assays previously (Boynton et al. 2007). We prepared PBMCs by Ficoll-Hypaque separation and froze cells in 30% fetal calf serum, 60% RPMI medium, and 10% dimethyl sulfoxide (Gibco, Gaithersburg, MD). We included cells from two control subjects in every run.

For the ^3^H-thymidine incorporation, we incubated PBMCs in microtiter plates with PHA of 0.1 and 0.5 μg/mL in five replicates each. After incubation for 3 days at 37°C, we pulsed the cells for 24 hr with ^3^H-thymidine and then harvested and counted the cells with a β-counter. We express the PHA-stimulated lymphocyte proliferation index as counts per minute of stimulated cells divided by counts per minute of unstimulated cells.

For the cell-division tracking method, we added carboxy-fluorescein diacetate succin-imidyl ester (Molecular Probes), a precursor of carboxy-fluorescein succinimidyl ester suspension at a final concentration of 10 μM. We incubated, washed twice, and resuspended the cells and then pipetted them into 16 wells of a microtiter plate (100,000 cells/well). Next, we added anti-CD3 antibody (BD Biosciences, San Jose, CA) to eight of the wells to specifically stimulate T lymphocytes. We used the remaining eight wells as control unstimulated cells. After incubation for 3 days at 37°C, we pooled identical wells and incubated the cells for 3 more days. On the sixth day, we harvested the cells and measured the CFSE–fluorescein isothiocyanate intensity of viable lymphocytes with a flow cytometer (XL-MCL; Beckman Coulter, Miami, FL).

We report four variables that quantify anti-CD3–stimulated lymphocyte proliferation measured by the cell-division tracking method: *a*) The proliferation index is the ratio of the total number of cells (parent and newly proliferated) to the number of back-calculated original parent cells; *b*) the parent percent represents the percentage of cells in the source cell population that did not divide; *c*) the precursor frequency represents the fraction of cells from the source population (i.e., parent cells) that divided three or more times; and *d*) the upper generational proliferation index is the ratio of the total number of cells (parent and newly proliferated from generations three and above) to the number of back-calculated original parent cells (Boynton et al. 2007).

### Geocoding

We geocoded subjects’ residences at the study baseline using ArcGIS software (version 9.0) and StreetMapUSA data (2004; both software and data from Environmental Systems Research Institute, Redlands, CA).

### Traffic proxy measures

**Figure 1:**
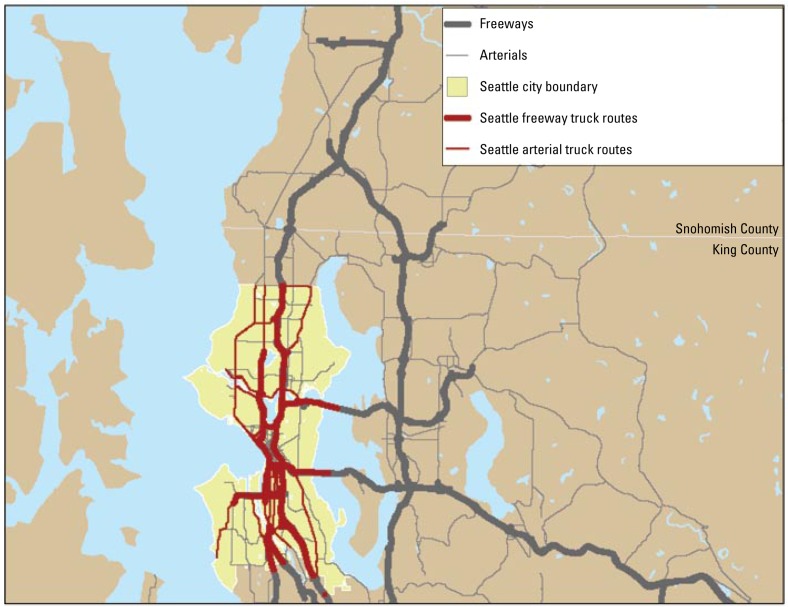
Freeways, arterials, and truck routes in the study area (freeway and principal street shapefiles created by King County; truck route shapefile created by the City of Seattle) (University of Washington 2007).

We obtained 2005 data on road networks through the Washington State Geospatial Data Archive (University of Washington 2007). Freeways are defined as limited-access highways (Haff 1993). Arterials are major roads between communities, population centers, and facilities. Truck routes are state routes or arterials designated by the city of Seattle as accommodating “significant freight movement” (Seattle Department of Transportation 2007); these data were available only for the city of Seattle. Freeway, arterial, and truck route road networks are shown in Figure 1.

We measured the distance from the subject’s residence to the nearest road of each particular type using ArcGIS. We computed several traffic proxy variables for each road type. A dichotomous variable indicated the presence of a particular type of road within 150 m of the subject’s residence. We chose the distance of 150 m for the “at-risk” category *a priori* based on findings from previous research that suggest that pollutants generated from on-road vehicles drop off exponentially with distance from the edge of a road, and that these vehicle-generated pollutants compose only a small fraction of the total pollutants at > 150 m (Zhu et al. 2002). However, we experimented with cut points of 100 and 200 m for the “at-risk” distance to evaluate the sensitivity of our results to the cut point chosen. We also created a set of indicator variables for categories of increasing residential proximity to the nearest road of each particular type (categories of > 300–500 m, > 200–300 m, > 150–200 m, > 100–150 m, > 50–100 m, and 0–50 m, with > 500 m as the referent).

### Census covariates

We extracted census tract variables from the 2000 Census Summary File 3 (U.S. Census Bureau 2002), including variables describing the racial and ethnic composition, unemployment rate, median income, education level, housing tenure rate, and population and housing density of the residential census tract for each subject.

### Statistical analysis

We performed all statistical analysis using SAS version 9.0 (SAS Institute Inc., Cary, NC). Because of skewed distributions, we transformed CRP and SAA data using the natural logarithm, and IL-6 using the natural logarithm of the observed value plus 0.5.

We used linear regression analysis to investigate associations between each of the traffic proxy metrics and each of the immune outcomes, adjusting for potential confounding factors. We considered CRP, SAA, IL-6, and T-lymphocyte proliferation stimulated by PHA or anti-CD3 as independent outcomes. We considered NK cytotoxicity as a nonindependent repeated measure in generalized estimating equations (GEEs) using the two intermediate E:T ratio dilutions: 12.5:1 and 25:1; these two dilutions had the greatest reproducibility and were in the linear range (Shade et al. 2004). For the dichotomous traffic proxy variables for residence within 150 m of a particular road type, we present model-adjusted least squares means and 95% confidence intervals (CIs) for each category, generated from the lsmeans statement of Proc GENMOD in SAS.

We selected variables *a priori* for adjustment as potential confounders, including age, BMI, education (individual-level), season of enrollment, time spent outdoors, and median income in the census tract of residence. We investigated associations of the traffic proxies with the immune outcomes among several pairs or trios of subgroup strata: *a*) overweight (BMI < 30) versus obese (BMI ≥ 30) subjects; *b*) low (< $35,000) versus middle ($35,000 to $75,000) versus high (> $75,000) income; and *c*) subjects reporting an hour or more of exercise near traffic versus subjects reporting no exercise near traffic in the previous 3 months on the baseline questionnaire.

## Results

### Study population

The 115 women in the study were highly educated, mostly non-Hispanic white, with high BMI; almost half the study population was classified as obese (BMI ≥ 30 kg/m^2^) (Table 1); 27% (*n* = 31) of women in the study lived near (within 150 m) major arterials, 3% (*n* = 3) lived near freeways, and 15% (*n* = 9) of the 61 residents of Seattle, where truck routes were assessed, lived near designated truck routes. Women who lived near arterials were more likely to be younger, nonwhite, and less educated than those who did not and were more likely to live in census tracts in which the median income was lower; however, only the difference in census tract–level income was statistically significant (Table 1). Women who lived near arterials reported similar amounts of time spent outdoors compared with women who did not live near arterials. Table 2 shows the distribution of each immune outcome and the number of subjects with data for inclusion in analyses. We did not have adequate power to assess the effect of living near freeways, because only three subjects lived within 150 m of a freeway (data not shown).

We observed a statistically significant association, in our hypothesized direction of effect, between residence near arterials and NK cytotoxicity, within our *a priori* hypothesized at-risk group of women living within 150 m of major roads (Table 3). Those who lived near arterials had, on average, NK cyto toxicity (expressed as the percentage of cells killed) of 19.5% (95% CI, 15.6–23.4%), compared with 24.8% (95% CI, 22.0–27.5%) among those who did not live near arterials. NK cytotoxicity was also lower among those living near truck routes than among those who did not, although this was not significant at a two-sided α level of 0.05. These associations were not sensitive to our *a priori* choice of covariates for adjustment of potential confounding; for example, models without inclusion of the covariates generated similar estimates for the mean NK cytotoxicity among those living near arterials (19.3%; 95% CI, 15.5–23.0%) and those living farther away (24.8%; 95% CI, 22.1–27.6%). The association between NK cytotoxicity and living near arterials was limited to those women who reported exercising near traffic (Table 4) or among women in the middle group of individual income ($35,000 to $75,000) (data not shown).

We observed significant associations of NK cytotoxicity with living near arterials when using alternate cut points of either 100 or 200 m for our definition of proximity to traffic, and the magnitudes of the associations were similar to our main analysis, indicating that this result was not excessively sensitive to our *a priori* cut point of 150 m. Residential proximity to truck routes was not significantly associated with NK cytotoxicity in the analysis using 100 m as the alternate cut point, and the association was strongest when using the cut point of 200 m. Estimated NK cytotoxicity was 15.5% among women living within 200 m of truck routes compared with 26.2% among women living at greater distances, and this difference was highly significant (*p* = 0.0009; data not shown).

There were no trends of association with increasing proximity to major roads for either road type, in models including indicator variables for categories of distance from a major road compared with a reference category of > 500 m [Supplemental Material, Table 1 (http://www.ehponline.org/members/2008/11580/suppl.pdf)].

We observed no associations between the markers of inflammation (CRP, SAA, IL-6) or PHA-stimulated lymphocyte proliferation and the traffic proxy variables (Table 5). We noted a significantly lower average upper generational proliferation index—one of the four measures of anti-CD3–stimulated lymphocyte proliferation—associated with residence near major arterials (Table 5). The other three measures of anti-CD3–stimulated lymphocyte proliferation were not associated with residence near arterials (Table 5), nor was residence near truck routes associated with any of the anti-CD3–stimulated lymphocyte proliferation measures (data not shown).

## Discussion

Ours is the first study, to our knowledge, of the association of traffic proxy measures and functional assays of cellular immunity. We observed an internally consistent association between residence within 150 m of major arterials or truck routes and lower NK cytotoxicity. The association was limited to those who reported exercising outdoors near traffic—individuals who presumably had higher exposure to traffic-related pollutants. We also found an association of living near arterials with lower than average lymphocyte proliferation; however, this result was not internally consistent among the various traffic proxy variables or lymphocyte proliferation measures, and we therefore consider the association tenuous. We observed no association between the traffic proxies and inflammation markers. In our modest-sized study, we analyzed five assays of immune status and several traffic proxy variables; therefore, our statistically significant results should be cautiously interpreted with respect to the possibility of chance findings and according to the consistency and robustness of the results.

NK cells are involved in the innate immune response and can destroy virally infected and transformed cells (Janeway et al. 2005). Although the relationship between *in vivo* NK function and NK cytotoxicity is unclear, low NK cytotoxicity has been associated with increased risk of infection, increased cancer risk, and increased risk of death among cancer survivors and institutionalized elderly (Albers et al. 2005; Imai et al. 2000; Kondo et al. 2003; Levy et al. 1991; Ogata et al. 2001). Researchers have found some evidence of associations between air pollution from traffic and infections (Brauer et al. 2002, 2006, 2007; Fusco et al. 2001; Lin et al. 2005; Morgenstern et al. 2007; U.S. EPA 2002), and associations between air pollution and NK cytotoxicity would strengthen the evidence for this important clinical outcome by suggesting a potential underlying biologic mechanism. The studies relating measures of ambient or traffic-related air pollution to colds and other infections have been conducted mostly among young children, and further research among older adults is needed to understand the possible health implications of the association we observed between reduced NK cytotoxicity and proximity to traffic.

In contrast to our results for NK cytotoxicity, we observed no consistent associations between traffic proxies and lymphocyte proliferation measures. As a predictor, lower *in vitro* lymphocyte proliferation has been found to be correlated with worse HIV prognosis (Hofmann et al. 1989; Schellekens et al. 1990), and we would expect that a human whose lymphocytes experience a decreased ability to proliferate upon stimulation would have increased vulnerability to infection. Nevertheless, it is not known whether an increased proliferative response in the *in vitro* lymphocyte proliferation assay corresponds with a more effective physiologic immune response (Vedhara et al. 1999). Measures from lymphocyte proliferation assays have proven to be less sensitive than NK cytotoxicity assays in nutrition intervention studies, indicating potentially lower sensitivity of lymphocyte proliferation to detect *in vivo* effects from external exposures (Albers et al. 2005).

On the basis of previous studies, we hypothesized an association between proximity to traffic and increased levels of CRP. About half of the previous studies of air pollution and CRP have observed associations between CRP and at least one measure of air pollution (usually a specific pollutant, e.g., fine or coarse PM) (Delfino et al. 2008; Diez Roux et al. 2006; Dubowsky et al. 2006; Peters et al. 2001; Pope et al. 2004; Riediker et al. 2004; Ruckerl et al. 2006; Seaton et al. 1999; Yue et al. 2007; Zeka et al. 2006); the prior evidence for associations of specific air pollutants with other inflammation markers, such as SAA or IL-6, is limited and conflicting (Delfino et al. 2008; Diez Roux et al. 2006; Frampton et al. 2004; Ghio et al. 2003; Gong et al. 2003; Seaton et al. 1999). There are several differences between our exposure metric and the studies that found an association with CRP. In the previous studies, higher CRP levels were associated with air pollution levels during a very recent time period such as the same day or preceding 1–9 days. Our exposure metric reflects an average exposure to local traffic-related pollutants in which short-term fluctuations due to background urban air pollution or personal exposures are not captured. It is possible that CRP levels (and other inflammatory markers) change in response to recent exposures in the preceding days or weeks, in which case our metric of average exposure would not be appropriate for modeling the association. Second, proximity to major roads represents the potential for mixed pollutant exposure from traffic. If the inflammatory response is specific to a certain pollutant such as PM, then the nonspecificity of our exposure metric may have obscured any association.

The strengths of this study include the objective outcome assessment using bio-markers, the strict criteria for blood draws, use of state-of-the-art assays for characterizing immune function, objective exposure assessment using a geographic information system (GIS), and the homogeneity of the study population. Our study also had several limitations. Although assigning exposure based on proximity to major roads is a straightforward approach to evaluating exposure to a pollution source, it is not as sophisticated as other approaches such as dispersion modeling. Measurement campaigns have indicated that although proximity measures are correlated with traffic pollutants, mis-classification occurs due to varying terrain and meteorologic conditions (Jerrett et al. 2005); therefore, our traffic proxies are subject to misclassification that may have caused spurious results. The proximity metric we employed may also be more vulnerable to confounding by socioeconomic factors or traffic-related noise than would more specific air pollutant exposure models. Nevertheless, adjustment for multiple individual-level and census tract–level socioeconomic variables did not change the associations we observed.

In this article, we report an association between residential proximity to traffic and an important *in vitro* marker of cellular immunity. Our study population was selected to be overweight or obese, but otherwise healthy. Approximately two-thirds of U.S. women in the age group we studied fall into this category (Mokdad et al. 2003; Ogden et al. 2006), and if the associations we observed are true, they would be relevant for a large proportion of the population. Additional studies of clinically relevant immune events such as infections and colds in relation to traffic-related pollution are needed to clarify the impact of traffic on the immune system and inform local land use policy.

## Correction

The values for the two rows “Proliferation index—PHA” in Table 5 were incorrect in the manuscript originally published online. They have been corrected here.

## Figures and Tables

**Table 1:** Study population characteristics.

Characteristic	All subjects (*n* = 115)	Not near arterial^a^ (*n* = 84)	Near arterial^a^ (*n* = 31)	*p*-Value^b^
Age, years [no. (%)]				
50–55	33 (29)	23 (27)	10 (32)	0.38
56–60	33 (29)	24 (29)	9 (29)	
61–65	16 (14)	13 (15)	3 (10)	
66–70	16 (14)	14 (17)	2 (6)	
71–75	17 (15)	10 (12)	7 (23)	
Mean ± SD	60.7 ± 6.8	60.6 ± 6.7	61.1 ± 7.3	0.55
BMI [no. (%)]				
24 to < 30	63 (55)	46 (55)	17 (55)	0.99
30 to < 35	37 (32)	27 (32)	10 (32)	
≥ 35	15 (13)	11 (13)	4 (13)	
Mean ± SD	30.3 ± 3.9	30.3 ± 3.9	30.2 ± 3.9	0.95
Race [no. (%)]^c^				
White (not of Hispanic origin)	101 (89)	75 (90)	26 (84)	0.33
Nonwhite	13 (11)	8 (10)	5 (16)	
Education [no. (%)]				
High school or less	17 (15)	13 (15)	4 (13)	0.54
Some college or college degree	57 (50)	39 (46)	18 (58)	
Graduate degree	41 (36)	32 (38)	9 (29)	
Season of enrollment [no. (%)]				
Winter	23 (20)	18 (21)	6 (19)	0.96
Spring	44 (38)	31 (37)	12 (39)	
Summer	23 (20)	16 (19)	7 (23)	
Fall	25 (22)	19 (23)	6 (19)	
Time spent outdoors [no. (%)]^c^				
0–1 hr/week	17 (15)	13 (15)	4 (13)	0.97
2 hr/week	24 (21)	18 (21)	6 (19)	
3–5 hr/week	29 (25)	21 (25)	8 (26)	
6 or more hr/week	45 (39)	32 (38)	13 (42)	
Median income of the census tract of residence (mean ± SD × 1,000)	$58 ± $18	$62 ± $19	$50 ± $14	0.04

a “Near arterial”: an arterial road was located within 150 m of a woman’s residence.

b For continuous variables, the *p*-value is from a *t*test (assuming equal variances) comparing the variable values between those living near arterials and those not-living near arterials; for categorical variables, the *p*-value is from a Pearson chi-square test of independence.

c Numbers not equal to total because of missing data.

**Table 2:** Immune biomarker distributions in the study population.

Biomarker	No.^a^	Mean ± SD	Minimum	Maximum
Inflammation markers				
CRP (mg/L)	114	3.7 ± 3.5	0.2	23.3
SAA (mg/L)	114	6.2 ± 5.5	1.4	43.0
IL-6 (pg/mL)	115	3.3 ± 3.1	0	20.0
NK cytotoxicity (%)				
25:1 E:T ratio	114	26.9 ± 13.3	4.35	68.9
12.5:1 E:T ratio	114	19.8 ± 12.0	3.15	59.6
PHA-stimulated lymphocyte proliferation				
Proliferation index^b^—PHA 0.1 μg/Ml	110	76.8 ± 47.5	2.1	209.5
Proliferation index^b^—PHA 0.5 μg/mL	110	207.3 ± 95.9	2.4	457.4
Anti-CD3–stimulated lymphocyte proliferation				
Proliferation index^c^	93	4.9 ± 2.0	1.3	11.8
Precursor frequency^d^	93	0.3 ± 0.1	0.02	0.5
Parent percent (%)^e^	93	15.0 ± 11.7	4.4	71.9
Upper generation proliferation index^f^	93	15.3 ± 5.2	7.3	31.7

a The number of women with nonmissing data for each assay is shown.

b Counts per minute of stimulated cells/counts per minute of unstimulated cells.

c Total number of cells (parent and newly proliferated) divided by the number of back-calculated original parent cells

d Fraction of cells from the source population (i.e., parent cells) that divided three or more times.

e Percentage of cells in the source cell population that did not divide.

f Total number of cells (parent and newly proliferated from generations three and above) divided by the number of back-calculated original parent cells.

**Table 3:** NK cytotoxicity by residence near (within 150 m) different major road types.

Residence	No.	NK cytotoxicity (%) [mean (95% CI)]
Residence near arterial		
No	84	24.8 (22.0–27.5)
Yes	30	19.5 (15.6–23.4)
Difference between means		*p* = 0.047
Residence near truck route^a^		
No	51	25.5 (22.1–28.9)
Yes	9	17.0 (10.9–23.1)
Difference between means		*p* = 0.06

For each major road type, we present model-adjusted least squares means of NK cytotoxicity at 12.5:1 and 25:1 E:T ratios generated from the lsmeans statement in proc GENMOD using a generalized estimating equation regression, while accounting for within-person correlation. The *p*-values represent an analysis of variance test of the difference between means. Estimates are adjusted for covariates selected *a priori*: age, BMI, education, blood draw season, time spent outdoors, and median income of residential census tract.

a Truck routes were classified only for the City of Seattle, where 61 women lived, and 60 of these women had non-missing data for NK cytotoxicity.

**Table 4:** NK cytotoxicity by residence near (within 150 m) different major road types and exercise near traffic.

	NK cytotoxicity (%)					
	No exercise near traffic			Any exercise near traffic		
Residence	No.	Mean	(95% CI)	No.	Mean	(95% CI)
Near arterial^a^						
No	51	21.4	(18.6–24.1)	26	28.8	(23.8–33.8)
Yes	11	21.0	(13.6–28.5)	15	20.7	(14.8–26.4)
Difference between means		*p* = 0.93			*p* = 0.08	
Near truck route^a^						
No	23	21.1	(17.7–24.4)	22	29.7	(26.0–33.4)
Yes	3	24.6	(13.1–36.1)	6	10.2	(2.9–17.5)
Difference between means		*p* = 0.58			*p* < 0.01	

For each major road type, we present model-adjusted least squares means of NK cytotoxicity at 12.5:1 and 25:1 E:T ratios generated from the lsmeans statement in proc GENMOD using a generalized estimating equation regression, while accounting for within-person correlation. The *p*values represent an analysis of variance test of the difference between-means. Estimates are adjusted for covariates selected *a priori*: age, BMI, education, blood draw season, time spent outdoors, and median income of residential census tract.

a Nonmissing data on both NK cytotoxicity and exercise near traffic were available for 103 women in the study area, and for 54 women in the City of Seattle where truck routes were assessed.

**Table 5:** Inflammation and lymphocyte proliferation measures by residence near (within 150 m) arterial road (estimated mean and 95% CIs).

Measure	Near arterial		Difference between means
	No (*n* = 84)^a^	Yes (*n* = 31)	
Inflammation markers	*n* = 83	*n* = 31	
CRP (mg/mL)	2.5 (2.1–3.0)	2.3 (1.6–3.1)	*p* = 0.62
IL-6 (pg/mL)^b^	2.4 (2.0–2.9)	2.1 (1.5–2.9)	*p* = 0.51
SAA (mg/mL)	4.9 (4.3–5.5)	5.3 (4.3–6.6)	*p* = 0.52
PHA-stimulated lymphocyte proliferation	*n* = 80	*n* = 30	
Proliferation index^c^—PHA 0.1 μg/mL	75.2 (64.7–85.8)	81.2 (63.6–98.7)	*p* = 0.57
Proliferation index^c^—PHA 0.5 μg/mL	206.9 (185.0–228.8)	208.4 (172.0–244.9)	*p* = 0.94
Anti-CD3–stimulated lymphocyte proliferation	*n* = 66	*n* = 27	
Proliferation index^d^	4.9 (4.4–5.4)	4.7 (4.0–5.5)	*p* = 0.68
Parent percent^e^	17.3 (14.3–20.3)	16.2 (11.5–21.0)	*p* = 0.71
Precursor frequency^f^	0.26 (0.23–0.28)	0.28 (0.24–0.32)	*p* = 0.32
Upper generation proliferation index^g^	16.0 (14.8–17.2)	13.5 (11.7–15.5)	*p* = 0.04

For each major road type, we present model-adjusted least squares means of each immune measure generated fom the lsmeans statement in proc GENMOD. The *p*-values represent an analysis of variance test of the difference between means. Estimates are adjusted for covariates selected *a priori*: age, BMI, education, blood draw season, time spent outdoors, and median income of residential census tract.

a Numbers reflect the distribution of the complete study population of 115 women; the number of women with nonmissing data for each assay varies, as shown.

b There were no missing data for the IL-6 assay; *n* = 115.

c Counts per minute of stimulated cells/counts per minute of unstimulated cells.

d Total number of cells (parent and newly proliferated) divided by the number of back-calculated original parent cells.

e Percentage of cells in the source cell population that did not divide.

f Fraction of cells from the source population (i.e., parent cells) that divided three or more times.

g Total number of cells (parent and newly proliferated from generations three and above) divided by the number of back-calculated original parent cells.

## References

[b1-ehp-117-373] Albers R, Antoine JM, Bourdet-Sicard R, Calder PC, Gleeson M, Lesourd B (2005). Markers to measure immunomodulation in human nutrition intervention studies. Br J Nutr.

[b2-ehp-117-373] Boynton A, Neuhouser ML, Wener MH, Wood B, Sorensen B, Chen-Levy Z (2007). Associations between healthy eating patterns and immune function or inflammation in overweight or obese postmenopausal women. Am J Clin Nutr.

[b3-ehp-117-373] Brauer M, Gehring U, Brunekreef B, de Jongste J, Gerritsen J, Rovers M (2006). Traffic-related air pollution and otitis media. Environ Health Perspect.

[b4-ehp-117-373] Brauer M, Hoek G, Smit HA, de Jongste JC, Gerritsen J, Postma DS (2007). Air pollution and development of asthma, allergy and infections in a birth cohort. Eur Respir J.

[b5-ehp-117-373] Brauer M, Hoek G, van Vliet P, Meliefste K, Fischer PH, Wijga A (2002). Air pollution from traffic and the development of respiratory infections and asthmatic and allergic symptoms in children. Am J Respir Crit Care Med.

[b6-ehp-117-373] Brook RD, Franklin B, Cascio W, Hong Y, Howard G, Lipsett M (2004). Air pollution and cardiovascular disease: a statement for healthcare professionals from the Expert Panel on Population and Prevention Science of the American Heart Association. Circulation.

[b7-ehp-117-373] Delfino RJ, Staimer N, Tjoa T, Polidori A, Arhami M, Gillen DL (2008). Circulating biomarkers of inflammation, anti-oxidant activity, and platelet activation are associated with primary combustion aerosols in subjects with coronary artery disease. Environ Health Perspect.

[b8-ehp-117-373] Devalia JL, Bayram H, Rusznak C, Calderon M, Sapsford RJ, Abdelaziz MA (1997). Mechanisms of pollution-induced airway disease: *in vitro* studies in the upper and lower airways. Allergy.

[b9-ehp-117-373] Diez Roux AV, Auchincloss AH, Astor B, Barr RG, Cushman M, Dvonch T (2006). Recent exposure to particulate matter and C-reactive protein concentration in the multi-ethnic study of atherosclerosis. Am J Epidemiol.

[b10-ehp-117-373] Dubowsky SD, Suh H, Schwartz J, Coull BA, Gold DR (2006). Diabetes, obesity, and hypertension may enhance associations between air pollution and markers of systemic inflammation. Environ Health Perspect.

[b11-ehp-117-373] Ershler WB (1993). Interleukin-6: a cytokine for gerontologists. J Am Geriatr Soc.

[b12-ehp-117-373] Frampton MW, Utell MJ, Zareba W, Oberdorster G, Cox C, Huang LS (2004). Effects of exposure to ultrafine carbon particles in healthy subjects and subjects with asthma. Res Rep Health Eff Inst.

[b13-ehp-117-373] Fusco D, Forastiere F, Michelozzi P, Spadea T, Ostro B, Arca M (2001). Air pollution and hospital admissions for respiratory conditions in Rome, Italy. Eur Respir J.

[b14-ehp-117-373] Ghio AJ, Hall A, Bassett MA, Cascio WE, Devlin RB (2003). Exposure to concentrated ambient air particles alters hematologic indices in humans. Inhal Toxicol.

[b15-ehp-117-373] Gong H, Sioutas C, Linn WS (2003). Controlled exposures of healthy and asthmatic volunteers to concentrated ambient particles in metropolitan Los Angeles. Res Rep Health Eff Inst.

[b16-ehp-117-373] Haff LJ (1993). King County Road Standards.

[b17-ehp-117-373] Hofmann B, Bygbjerg I, Dickmeiss E, Faber V, Frederiksen B, Gaub J (1989). Prognostic value of immunologic abnormalities and HIV antigenemia in asymptomatic HIV-infected individuals: proposal of immunologic staging. Scand J Infect Dis.

[b18-ehp-117-373] Imai K, Matsuyama S, Miyake S, Suga K, Nakachi K (2000). Natural cytotoxic activity of peripheral-blood lymphocytes and cancer incidence: an 11-year follow-up study of a general population. Lancet.

[b19-ehp-117-373] Janeway CA, Travers P, Walport M, Sholmchik MJ (2005). Immunobiology.

[b20-ehp-117-373] Jerrett M, Arain A, Kanaroglou P, Beckerman B, Potoglou D, Sahsuvaroglu T (2005). A review and evaluation of intraurban air pollution exposure models. J Expo Anal Environ Epidemiol.

[b21-ehp-117-373] Johnson BD, Kip KE, Marroquin OC, Ridker PM, Kelsey SF, Shaw LJ (2004). Serum amyloid A as a predictor of coronary artery disease and cardiovascular outcome in women: the National Heart, Lung, and Blood Institute-Sponsored Women’s Ischemia Syndrome Evaluation (WISE). Circulation.

[b22-ehp-117-373] Kondo E, Koda K, Takiguchi N, Oda K, Seike K, Ishizuka M (2003). Preoperative natural killer cell activity as a prognostic factor for distant metastasis following surgery for colon cancer. Dig Surg.

[b23-ehp-117-373] Kritchevsky SB, Cesari M, Pahor M (2005). Inflammatory markers and cardiovascular health in older adults. Cardiovasc Res.

[b24-ehp-117-373] Lebret E, Briggs D, van Reeuwijk H, Fischer P, Smallbone K, Harssema H (2000). Small area variations in ambient NO_2_ concentrations in four European areas. Atmos Environ.

[b25-ehp-117-373] Levy SM, Herberman RB, Lee J, Whiteside T, Beadle M, Heiden L (1991). Persistently low natural killer cell activity, age, and environmental stress as predictors of infectious morbidity. Nat Immun Cell Growth Regul.

[b26-ehp-117-373] Lin M, Stieb DM, Chen Y (2005). Coarse particulate matter and hospitalization for respiratory infections in children younger than 15 years in Toronto: a case-crossover analysis. Pediatrics.

[b27-ehp-117-373] McTiernan A, Ulrich CM, Yancey D, Slate S, Nakamura H, Oestreicher N (1999). The Physical Activity for Total Health (PATH) Study: rationale and design. Med Sci Sports Exerc.

[b28-ehp-117-373] Mizutani Y, Okada Y, Terachi T, Yoshida O (1996). Prognostic significance of circulating cytotoxic lymphocytes against autologous tumors in patients with bladder cancer. J Urol.

[b29-ehp-117-373] Mokdad AH, Ford ES, Bowman BA, Dietz WH, Vinicor F, Bales VS (2003). Prevalence of obesity, diabetes, and obesity-related health risk factors, 2001. JAMA.

[b30-ehp-117-373] Morgenstern V, Zutavern A, Cyrys J, Brockow I, Gehring U, Koletzko S (2007). Respiratory health and individual estimated exposure to traffic-related air pollutants in a cohort of young children. Occup Environ Med.

[b31-ehp-117-373] Ogata K, An E, Shioi Y, Nakamura K, Luo S, Yokose N (2001). Association between natural killer cell activity and infection in immunologically normal elderly people. Clin Exp Immunol.

[b32-ehp-117-373] Ogden CL, Carroll MD, Curtin LR, McDowell MA, Tabak CJ, Flegal KM (2006). Prevalence of overweight and obesity in the United States, 1999–2004. JAMA.

[b33-ehp-117-373] Peters A, Frohlich M, Doring A, Immervoll T, Wichmann HE, Hutchinson WL (2001). Particulate air pollution is associated with an acute phase response in men; results from the MONICA- Augsburg Study. Eur Heart J.

[b34-ehp-117-373] Pope CA, Hansen ML, Long RW, Nielsen KR, Eatough NL, Wilson WE (2004). Ambient particulate air pollution, heart rate variability, and blood markers of inflammation in a panel of elderly subjects. Environ Health Perspect.

[b35-ehp-117-373] Riediker M, Cascio WE, Griggs TR, Herbst MC, Bromberg PA, Neas L (2004). Particulate matter exposure in cars is associated with cardiovascular effects in healthy young men. Am J Respir Crit Care Med.

[b36-ehp-117-373] Roorda-Knape MC, Janssen NA, de Hartog J, van Vliet PH, Harssema H, Brunekreef B (1999). Traffic related air pollution in city districts near motorways. Sci Total Environ.

[b37-ehp-117-373] Ruckerl R, Ibald-Mulli A, Koenig W, Schneider A, Woelke G, Cyrys J (2006). Air pollution and markers of inflammation and coagulation in patients with coronary heart disease. Am J Respir Crit Care Med.

[b38-ehp-117-373] Schellekens PT, Roos MT, De Wolf F, Lange JM, Miedema F (1990). Low T-cell responsiveness to activation via CD3/TCR is a prognostic marker for acquired immunodeficiency syndrome (AIDS) in human immunodeficiency virus-1 (HIV-1)-infected men. J Clin Immunol.

[b39-ehp-117-373] Seaton A, Soutar A, Crawford V, Elton R, McNerlan S, Cherrie J (1999). Particulate air pollution and the blood. Thorax.

[b40-ehp-117-373] Seattle Department of Transportation (2007). Street Classification Maps.

[b41-ehp-117-373] Shade ED, Ulrich CM, Wener MH, Wood B, Yasui Y, LaCroix K (2004). Frequent intentional weight loss is associated with lower natural killer cell cytotoxicity in postmenopausal women: possible long-term immune effects. J Am Diet Assoc.

[b42-ehp-117-373] U.S. Census Bureau (2002). Census 2000 Summary File 3—Washington State.

[b43-ehp-117-373] U.S. EPA (2002). Health Assessment Document for Diesel Engine Exhaust EPA/600/8-90/057F.

[b44-ehp-117-373] University of Washington (2007). WAGDA (Washington State Geospatial Data Archive).

[b45-ehp-117-373] Vedhara K, Fox JD, Wang EC (1999). The measurement of stress-related immune dysfunction in psychoneuroimmunology. Neurosci Biobehav Rev.

[b46-ehp-117-373] White RH, Spengler JD, Dilwali KM, Barry BE, Samet JM (2005). Report of workshop on traffic, health, and infrastructure planning. Arch Environ Occup Health.

[b47-ehp-117-373] Yeh ET, Willerson JT (2003). Coming of age of C-reactive protein: using inflammation markers in cardiology. Circulation.

[b48-ehp-117-373] Yue W, Schneider A, Stolzel M, Ruckerl R, Cyrys J, Pan X (2007). Ambient source-specific particles are associated with prolonged repolarization and increased levels of inflammation in male coronary artery disease patients. Mutat Res.

[b49-ehp-117-373] Zeka A, Sullivan JR, Vokonas PS, Sparrow D, Schwartz J (2006). Inflammatory markers and particulate air pollution: characterizing the pathway to disease. Int J Epidemiol.

[b50-ehp-117-373] Zhu Y, Hinds WC, Kim S, Sioutas C (2002). Concentration and size distribution of ultrafine particles near a major highway. J Air Waste Manag Assoc.

